# Overcoming inherent problems of preference-based techniques for measuring health benefits: An empirical study in the context of kidney transplantation

**DOI:** 10.1186/1472-6963-6-3

**Published:** 2006-01-14

**Authors:** Nick Kontodimopoulos, Dimitris Niakas

**Affiliations:** 1 Faculty of Social Sciences, Hellenic Open University, Riga Fereou 169 & Tsamadou 262 22 Patra, Greece

## Abstract

**Background:**

Economic valuations of health care programs often require using patients as subjects, implying that research methodology should conform to the surrounding social, cultural and ethical context. The significance of patients' opinions in health care decisions has been well defined but in Greece, and perhaps elsewhere, clinicians remain skeptical. The purpose of this study was to investigate, for the first time in Greece, the feasibility of measuring preference-based health-state utilities and willingness to pay and to determine the context-based adaptations required to overcome inherent elicitation problems.

**Methods:**

A survey including a time trade-off (TTO), a standard gamble (SG), and two willingness-to-pay (WTP) questions was self-administered to a homogenous group of 606 end stage renal disease patients in 24 dialysis facilities throughout Greece and the overall response rate was 78.5%. Typical elicitation methods were adapted to overcome methodological problems such as subjective life expectancy and question framing. Spearman's correlation coefficients were calculated between utilities and WTP and parametric tests (independent samples t-test and ANOVA) examined score differences as a result of demographic and clinical factors.

**Results:**

Mean health-state utilities were 72.56 (TTO) and 91.06 (SG) and these were statistically significantly different (*P *< 0.0005). Significant correlations, in the expected directions, were observed between TTO – SG, TTO – WTP and SG – WTP (*P *< 0.01). High ceiling effects were observed in the TTO and SG methods indicating patients' adversity to risk and unwillingness to trade-off life years. Higher WTP was observed from younger patients (*P *< 0.0005), males (*P *< 0.05), higher education levels (*P *< 0.01), single (*P *< 0.0005) and employed (*P *< 0.005).

**Conclusion:**

This study demonstrated, to a fair extent, that adapting research methods to context-based particularities does not necessarily compromise results and should be considered in situations where standard methods cannot be applied. On the other hand, it is emphasized that the results from this study are preliminary and should be interpreted cautiously until further research demonstrates the practicality, reliability and validity of alternative measurement approaches.

## Background

Bioethics has identified the significance of the patient's opinion in health care decisions where facts known only by doctors have to be supplemented by values known only by patients. Pressures to improve the cost-effectiveness of medical care have increased interest in perceived health, health-related quality of life (HRQoL) and health-state utilities as ways of bringing health assessment closer to the patient's perspective. Despite conceptual and methodological difficulties, the assessment of (HRQoL) can assist in caring for chronically ill patients and the most common measuring approach is using descriptive psychometric instruments that describe health status in a number of different areas. However, in cost utility analyses, where it is desirable to measure overall HRQoL along a single cardinal scale, descriptive instruments provide limited information and health effects, in this case, are measured usually in terms of quality-adjusted life years (QALYs) [1].

There are three main preference-based methods for measuring QALY weights which are often referred to as health-state utilities: the rating scale (RS), the time trade-off (TTO) and the standard gamble (SG) [2]. Although the literature is rich in studies involving the elicitation of utilities in many health care problems, only limited information is available in the context of dialysis [3,4]. Contingent valuation (CV) is the commonly used direct technique for eliciting willingness-to-pay (WTP). It involves asking how much the respondent would be willing to pay for the good in question. Values are derived by setting up a hypothetical or contingent market for this good which cannot be directly observed in a real market place and the construction of this hypothetical market will influence the values derived [5]. The literature contains extensive reviews of CV studies in health care [6,7] but to our knowledge it has not been previously applied to dialysis patients.

It is clear that economic evaluations of health care programs often require the involvement of patients as subjects, therefore it is important that the applied methods are ethically sound and conform to existing social and cultural conditions [8]. Furthermore, medical staff often express methodological objections to studies they perceive as inappropriate for their patients. These objections often stem from the fact that physicians rely mostly on objective clinical assessments and are hesitant to involve patients in decision making processes [9]. In light of this, it is important to adapt study methods to context-specific barriers and overcome inherent application problems, which would otherwise hinder such economic evaluations.

This was the first study in Greece aiming to measure health-state utilities and willingness to pay in any health problem and the study population chosen was end stage renal disease (ESRD) patients on dialysis. This patient group was an attractive study option because the alternative of kidney transplantation could trigger their interest to hypothetically trade time and wealth for better health. The typical TTO, SG and WTP elicitation methods were adapted to population and context-specific cultural and ethical barriers. Given the overall uncertainty associated with modifications to any standard research methodology, the main objective of this study was to investigate the feasibility, rather than the reliability or validity, of cultural adaptations to the TTO, SG and WTP methods. It was hypothesized that SG and TTO would positively correlate, SG would produce higher scores, and both utilities would negatively correlate with WTP in accordance to similar studies involving other patient groups [10-13].

### Health system and renal replacement therapy in Greece

A public-private mix for both funding and delivery currently characterizes the health care system in Greece. It is financed by a combination of tax-based and insurance-based statutory financing, supplemented by voluntary funds, with the latter accounting for approximately 40% of overall financing. The state budget, financed through taxation at the central level only, is responsible for financing rural health center and rural clinic expenditures, salaries of personnel in public hospitals, subsidies of public hospitals (involving payments to hospitals over and above the per diem fees paid by the health insurance funds), subsidies of the social insurance funds, and subsidies of civil servant health insurance, capital investments, public health, medical education, etc. As for compulsory health insurance there are approximately 30 funds, which cover the bulk of the population. Membership is compulsory and based on occupation. Most of these funds are public entities and, while autonomous, they operate under extensive control by the central government and are responsible for financing hospitals on a per diem basis [14].

Currently, 9,500 patients are on some form of renal replacement therapy (RRT) with approximately 75% on long-term dialysis, 8% on peritoneal dialysis and the others have been transplanted. The augmentation of the RRT "pool" (taking into account patient deaths) is close to 8% per annum. Regarding cost, dialysis is one of the most expensive techniques in modern substitutive medicine. If we are to accept the Greek estimate of €240 for the average cost of a single dialysis session, then its aggregate economic impact exceeds 250 million Euros per annum in Greece. Of this, less than 10% is spent on technology, while personnel account for the greatest part of the cost [15]. Access to dialysis is free in both public and private facilities and expenses are covered by health insurance. The cost of a kidney transplantation, according to a recent (2003) estimation by the Hellenic National Transplant Organization, is approximately €22,500.

## Methods

### Typical elicitation

In the SG method, QALY weights for health states are determined by comparing a specific number of years in health state *Hi *with a gamble (a treatment) offering two reference outcomes, which are a probability *p *of full health for the same number of years and a probability *1-p *of immediate death. The probability *p *of full health is varied until the respondent is indifferent between the two alternatives. The indifference probability is the weight to be assigned to health state *H*_*i*. _On the other hand, the TTO method typically requires comparing *Y *years in a particular health state *H*_*i *_to *X *years in full health. The number *X *is varied until the respondent is indifferent between the alternatives. The QALY weight assigned to health state H_*i *_is then set equal to *X/Y*. The TTO and SG are choice-based techniques that differ significantly in that TTO is riskless whereas SG is framed in terms of risk and incorporates the respondent's attitude towards risk. According to *prospect theory*, attitudes towards risk consist of two components, one of which reflects sensitivity to outcomes and the other sensitivity towards chance [16].

On the other hand, WTP is the only benefit measure reflecting conventional microeconomic properties and requires respondents to consider a health-wealth tradeoff, making it a cognitively more demanding method. It imposes no restrictions on which attributes of a program can be considered in its valuation [17]. Anything, over which the individual has preferences, including a particular health outcome, is considered to be an "economic good". Similarly to the QALY approach, the value of reducing a specific mortality risk in the current period depends on life expectancy, competing mortality risk and the individuals's health if he/she is to survive the risk, baseline risk and on income or wealth. Furthermore, a person's WTP is clearly limited by his/her ability to pay [18] and it has been shown that WTP is positively related to income [19].

### Ethical and cultural barriers

In most facilities the review boards and clinical staff, due to perceived ethical considerations, refused to grant permission for the patients to be subjected to questions implying, even hypothetically, the age of death, i.e. the timeframe *Y *mentioned previously. Religious issues had to be considered also because the duration of life and time of death are perceived as "unknowns" which are controlled "from above" and not as tradable products. Furthermore, a specific timeframe, e.g. 10 or 20 years, could bias the utility scores if subjective expectations about the age of death were different [20]. In this study, the "gamble" was a kidney transplant and the outcomes were a probability *p *of full health and a probability *1-p *of immediate death with the latter again raising concern from clinical staff. Ethical implications were foreseen in that the immediate death probability *1-p *could generate false perceptions to patients about the actual risks associated with kidney transplants and this, in turn, might result in increased unwillingness from patients to undergo a transplant should they have the opportunity to do so in a real-life situation.

In the Greek health system, as well as in most others worldwide, the cost of dialysis treatments and kidney transplants is fully covered by the public sources of financing and there are no existing co-payments required from the patients. The ethical concern in the case of WTP was that patients might become severely misconceived in that they would be required to pay for a kidney transplant in a real life situation. Given the overall low educational level of the patients, many clinicians felt that the WTP questions could create a false impression about the existence of an underlying "black-market" for transplants where the wealthier and "better connected" could skip the waiting list and "purchase" a kidney transplant.

### Adapting study design

These objections had to be taken into account in designing the study. The first methodology adaptation involved offering the respondents health state H_*i*_, (their current health state) for the rest of their expected lifespan instead of a fixed number of years. The difficulty in calculating utilities, particularly in the case of TTO, came from the fact that patients' remaining life years were unknown as this is part of the contract of life. We adopted the *"fair innings" *argument according to which all people are entitled to a normal span of life [21] and encouraged patients to subjectively assume that dialysis would secure for them a number of life years similar to that of the general population. For calculation, we arbitrarily assumed life expectancy at 80 years of age without disclosing this information to the patients. Those already older or willing to tradeoff years which added to their current age, exceeded 80, were excluded. In the case of SG, the problem was less complicated since the fraction of remaining lifespan the individual would be willing to sacrifice to improve health does not depend on the remaining lifespan, a condition known as "*constant proportional tradeoff longevity for health" *[22].

In both SG and TTO, the health state to be assessed is compared to an alternative that can be framed either as a gain or a loss and it is well known, from many studies, that the type of framing affects behavior, especially in the case of the SG method [23]. The reason for this diversion may be loss aversion, meaning that if a change is perceived as a loss compared to a reference level, it results in a greater change in utility than if the same change is perceived as a gain. Since dialysis patients are already in a less than ideal health state it is easier for them to specify the maximum probability *1-p *of the unfavorable outcome rather than the minimum probability *p *of the favorable one. This particular adaptation of the SG elicitation methodology helped to overcome one more problem. Specifically, when outcomes are not certain but occur with known probabilities, people transform these probabilities into decision weights and in particular, they overestimate small probabilities and underestimate large probabilities [24].

Although the WTP methods used in this study are comparable to those undertaken in other countries, the actual elicitation techniques were chosen according to particularities of the sample and concerns from the physicians. Considering, once again, the low educational level of the respondents and that WTP studies are, to date, practically unknown in Greece, it was important that the elicitation format not create suspicions about the intent of the questions. For example, the bidding game, in which an auction process is simulated often resembling actual market situations, could make the patients uncomfortable and unwilling to participate or even make them question if health care is truly free. In any case, bidding games require interviewers or interactive computer programs and are therefore more costly than other methods, which can be carried out via self-administration.

In this study, WTP was measured using the dichotomous format followed by an open-ended question, a technique that has been shown to increase the statistical efficiency of the responses [25]. Patients were initially asked if they were willing to pay, out-of-pocket, €15,000 for a kidney transplant. This bid is close to the actual cost of a kidney transplant in Greece. It is unarguably a heavy economic burden for the average Greek, however it is realistic and, in most cases, could be raised with the help of the greater family or friends. The objective was for respondents to value the transplant in comparison to other personal and/or family needs and produce realistic answers. In view of this, they were advised to consider that paying this amount would imply that other needs might not be satisfied. It was emphasized once more that the situation was strictly hypothetical and no payments would be requested in a real situation.

**Figure 1:**
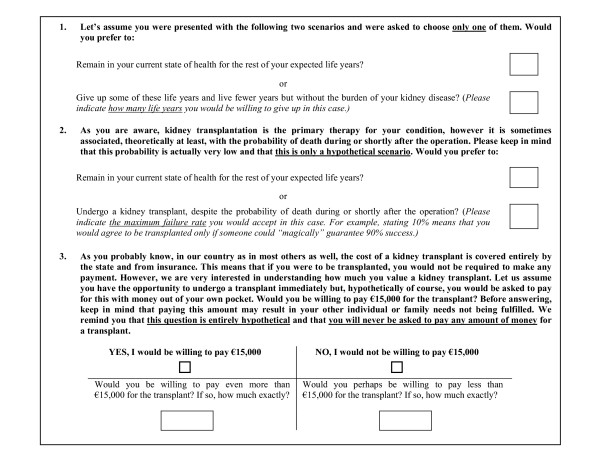
The time trade-off, standard gamble and willingness to pay questions.

In a subsequent open-ended question, patients responding "yes" to the dichotomous question were asked if they would be willing to pay more than €15,000 and, if so, to specify the exact amount. Those answering "no" were asked if they would be willing to pay a smaller amount and again to specify. In cases where only the dichotomous question was answered, the final WTP was taken as €15,000 for the "yes" respondents whereas the "no" respondents were taken as true zero bidders and included in the analysis. The actual questions asked in this study have been translated and are shown in Fig. 1.

### Sample and data collection

We randomly selected 25% of the dialysis facilities currently operating in Greece (32 out of 128) and requested permission to conduct the study using the standard elicitation methods. Each review board examined the survey and only three granted immediate permission without any methodological or ethical objections. The other twenty-nine perceived at least one of the problems mentioned previously. We reapplied with the adapted versions of elicitation, to overcome initial denial. This resulted in twenty-one more facilities agreeing to participate (24 in total – 75%), with the other eight remaining unconvinced and eventually excluded. The adapted versions of elicitation were used in all dialysis centers.

To facilitate a large population study without the need for costly interviewers, the questions were paper-based for self-administration, a technique that has been shown to be a reliable substitute to typical utility elicitation methods in the case of SG [26,27]. As for WTP, self-administration is once again a logical substitute to the otherwise preferred method of face-to-face interviews. However, the latter represents the most costly way of collecting data and, not surprisingly, in a review of 71 WTP surveys of health and health care published in English during the period of 1985-1998, only 27 (38%) employed face-to-face data interviews compared to 34 (47.9%) employing self-administration or post [28].

Each facility appointed a dialysis nurse who was trained to distribute the questionnaire, explain the purpose of the study and provide assistance when needed. Adult patients (aged 18+) were eligible for the study and were chosen by the clinical and nursing staff in each facility on the basis of their mental and physical ability to read, comprehend and complete the self-administered survey, with the least possible assistance. Others not fulfilling this criteria, e.g. minority groups or illiterates, were deemed unable to participate. In order to ensure informed consent, the patients were asked to read an accompanying letter emphasizing that participation was voluntary and anonymous and that only aggregate results would be reported. The survey consisted of common socio-demographic and clinical questions, TTO and SG utility questions and two WTP questions. On aggregate, 606 dialysis patients from the 24 participating facilities were deemed suitable candidates. The response rate was 78.5% with 504 patients eventually completing the survey over the period April 2004 – December 2004. Monetary values are reported in Euros for 2004 (1€ = 1.3 USD).

### Analysis

Spearman's correlation coefficients were used to analyze the direction and the strength of the relationship between the health-state utilities and willingness to pay. Parametric tests (independent samples t-test and ANOVA) were performed to examine differences in scores, for each preference-based utility measure and WTP, as a result of various socio-demographic and clinical factors such as age, sex, education, familial status, employment, comorbidities and previous unsuccessful kidney transplant and multiple linear regression analysis was performed to determine the most significant predictors. All analyses were performed using SPSS software, version 12.0 (SPSS Inc., Chicago IL).

## Results

Socio-demographic and clinical characteristics of the sample (N = 504) are given in Table 1. The majority of respondents were male (61.6%) and the mean age of the whole sample was 57.1 years. Almost half had completed only primary school and 14.9% percent had a university education. Most patients were married (67.8%) and currently employed (38.7%). The average time on dialysis treatment was 6.1 years and more than one third of the sample was on the transplant waiting list. One out of ten patients had already undergone an unsuccessful transplantation and had returned to dialysis. Almost a third had been hospitalized at least once over the past year for reasons attributed to ESRD and 41.9% reported suffering from at least one co-morbid condition.

The mean health-state utilities based on the TTO and SG methods were 72.56 and 91.06 respectively (Table 2), which were statistically significantly different (*P *< 0.0005). The utility scores were further examined with parametric tests (independent samples *t-*test and ANOVA) in which age, sex, education, family/occupational status and co-morbidities where used as grouping variables. Significant differences (*P *< 0.05) were observed only with age and interestingly older patients generated higher utilities with both methods. The high ceiling effects in the TTO and SG methods are indications of patients' adversity to risk and unwillingness to trade-off life years.

Concerning WTP, 40.5% of the respondents were unwilling to pay any amount of money for a kidney transplant, 9.7% would pay up to €15,000, 18.8% percent would pay exactly €15,000 and 31.0% would pay more, with €100,000 being the highest reported amount. The median WTP for all participants was €10,000 (see Table 3). We again examined mean WTP with independent samples *t-*tests and ANOVA in order to assess if the same socio-demographic and clinical factors were important determinants and interestingly we found that all, except co-morbidities, generated statistically significant differences. Higher WTP was observed from younger patients (*P *< 0.0005), males (*P *< 0.05), higher education levels (*P *< 0.01), single (*P *< 0.0005) and employed (*P *< 0.005). Multiple linear regression analysis, using a forward stepwise selection procedure, was performed and the best-fitting model incorporated age (*P *< 0.01) and family status (*P *< 0.05) as significant predictors of WTP, providing an explanatory power of 8.1%. The addition of various clinical and health related variables as predictors had little impact on the results.

It is also interesting that patients who had experienced an unsuccessful kidney transplant were willing to pay approximately 60% more than others who had never been transplanted (*P *< 0.05). Spearman's correlation coefficients between utility measures and willingness to pay are presented in Table 4. We observed a strong positive association between TTO and SG and strong negative correlations between WTP and the utility measures (*P *< 0.01). The latter implies that, as expected, higher utility scores, i.e. better quality of life, result in a reduced willingness to pay.

## Discussion

We applied utility elicitation and contingent valuation methods to a sample of 504 dialysis patients. This was the first study in Greece attempting to elicit WTP in any context and also the first involving SG and TTO to value health states. Previously, work has been performed in Greece to translate and validate the EQ-5D instrument, according to recommendations by the EuroQol Group. The results indicated that the instrument is valid and reliable and can be used effectively in quality of life measurement in Greek clinical trials and population-based exercises [29]. However, the overall experience of the Greek population with self-completion of health interviews is extremely limited and this constituted a major factor requiring attention in this study.

The methods used here could be characterized as "non-standard" in the sense that typical procedures, especially in the case of SG and TTO, were modified to overcome existing cultural and ethical barriers. The study demonstrated the feasibility of measuring health-state utilities and willingness to pay in this patient population, however it must be stressed that this does not ensure the validity of these methods and it is important to interpret the results cautiously. We must take into consideration that the reliability and validity of preference-based methods could vary in a cross-cultural context due to differences in the way an individual values health. This implies that the comparability of results to those from other countries is limited. Even more so in this case where methodological adaptations were employed.

In accordance to the literature, the methods yielded systematically different results [30]. The cognitive processes involved in eliciting patients' preferences for health outcomes can be quite onerous and are affected by framing effects, contexts, anchoring points, duration of conditions, time preferences and attitudes towards risk [31,32]. Furthermore, willingness to tradeoff life years or to gamble with life is dependent on how life and health are valued by the respondent [33]. The patients in this study were risk averse and unwilling to gamble and also unwilling to trade many life years, as suggested by the ceiling effects of TTO and SG scores. This could be due to religious factors or to the "coping mechanism" where patients gradually learn to adapt to their situation and, despite severe physical limitations, subjectively experience a relatively high HRQoL. On the technical side, the high TTO and SG scores could be due to framing the alternatives as a loss, resulting in patients favoring their present health state.

In an attempt to assess the validity of the measures, we could follow the axioms of von Neumann and Morgenstern, where the SG method has a basis in expected utility theory and is the standard technique for measuring decision making under uncertainty, implying that it gives a valid health-state utility by definition [34]. In this study, TTO showed a strong correlation with SG, therefore it must also be relatively valid. However, it has been argued that the SG results in a utility function that captures risk attitudes, which implies that if a decision involves uncertainty, the utility function should be used [35]. Providing the absence of a gold standard, preference measurements are value based, and hence, the weights used in cost-utility studies will remain questionable and controversial. This obviously applies to the present study as well.

Approximately 40% of the respondents were unwilling to pay any amount for a kidney transplant and interestingly 75% of these "unwilling" patients were not on the transplant list. Perhaps the awareness that they will not be transplanted in the future makes them unwilling to even hypothetically trade wealth for health. Indeed, the mean age of "unwilling" and "willing" patients was 61.8 and 53.9 years respectively and the differences statistically significant (*P *< 0.001). Almost 19% of all respondents were willing to pay exactly the price set in the dichotomous question, suggesting the existence of "yea-saying" bias, i.e. the tendency of some respondents always to answer "yes" irrespective of the price level [36-38]. We also observed that patients who had undergone an unsuccessful transplant were willing to pay approximately 60% more than those never transplanted (*P *< 0.05). A possible explanation is that experiencing the benefits of the transplant even briefly, makes them less hesitant to consider a second operation.

Willingness to pay was significantly (and negatively) correlated with both TTO and SG. This implies that higher utility scores result in a reduced willingness to pay, and this is both logical and expected, according to one of our initial hypotheses. However, it is important to keep in mind that the methods used to measure health-state utilities and willingness to pay are prone to large random errors which eventually decrease correlations. In this respect, an advantage of descriptive quality of life instruments is that they are easy to administer and understand which leads to lower random errors and higher reliability. Given these advantages, it would be interesting to be able to predict the results of health-state utility methods and the willingness to pay methods based on the results from quality of life instruments [39].

This study is not free from some limitations. We used an ex post approach and an actual patient population to assess WTP. While this approach is consistent with many previous CV studies in the health field [40], some have argued that WTP should be assessed ex ante from an insurance perspective in which people from the general population are informed about their own probabilities of needing a service, and are asked about their ex ante insurance premium payment, which focuses on option value [41]. While the ex ante approach is recommended in the health economics literature [42], the alternative approach utilized in our study has several practical advantages. For example, patients are better informed than the general population about the consequences of a treatment and therefore the CV questions should be easier to understand. However, the absolute WTP values obtained should be interpreted cautiously, since the current study may have over- or under-estimated true WTP. In any case, the aim was to investigate if correlations between WTP and health state utilities elicited from non-standard methods are as expected and, in this respect, the actual amounts patients are willing to pay are not really important.

For ethical reasons previously mentioned, we set the time frame as each patient's remaining lifetime and this could have added a disturbing influence on the TTO utilities, namely the effect of subjective expectations about the age of death and quality of life. Patients have their own beliefs about their expected age of death and future quality of life but these expectations could be very different even between patients of similar age and clinical condition and in most cases different from the clinically expected duration of life [43]. In this respect, the SG method seems to be advantageous as the time horizon specified in the question is assumed to be independent of the QALY-utility derived [44].

## Conclusion

We generally suggest that adaptive study designs should be considered in order to overcome methodological and ideological objections from patients, review boards or medical staff, but results should be examined cautiously and critically. This is especially true in a study such as the present involving methods, which are controversial even in their standard form and alterations to typical practice could create overall uncertainty. Despite this, our results appear to conform to certain expected and hypothesized patterns constituting them, at the very least, interesting. Given the scarcity of utility studies and the non-existence of WTP studies involving dialysis populations, further research is necessary in order to test adapted methodologies and, more importantly, the meaningfulness of results.

## Competing interests

The author(s) declare that they have no competing interests.

## Authors' contributions

NK was responsible for designing the study, acquiring and analyzing the data and drafting the manuscript. DN was responsible for conception of the study. Both authors contributed to interpreting the results and revising the manuscript for intellectual content and have read and approved the final manuscript.

## Pre-publication history

The pre-publication history for this paper can be accessed here:



## Figures and Tables

**Table 1:** Characteristics of the sample (N = 504)

**Demographics**	**N (% valid)**	**Clinical data**	**N (% valid)**
Gender (male)	308 (61.6)	Primary kidney disease	
Age (mean ± SD)	57.1 ± 14.9	Glomerulonephritis	109 (22.2)
Education		Polycystic kidney	60 (12.2)
Primary school	200 (42.6)	Hypertension	97 (19.8)
Secondary school	80 (17.1)	Diabetes	50 (10.2)
High school	119 (25.4)	Other	84 (17.2)
University	70 (14.9)	Unknown	90 (18.4)
Family status		Years on HD (mean ± SD)	6.1 ± 5.6
Single	95 (19.0)	On transplantation list	187 (37.6)
Married	339 (67.8)	Previous unsuccessful transplant	52 (10.3)
Divorced/Separated	27 (5.4)	Co morbidity	211 (41.9)
Widowed	39 (7.8)	Cardiovascular disease	91 (43.1)
Occupational status		Diabetes	46 (21.8)
Unemployed/Retired	226 (45.0)	Physical impairment	20 (9.5)
Employed	195 (38.7)	Other	54 (25.6)
Keeping house/Student	82 (16.3)	One or more renal disease attributed hospitalizations over the past year	152 (30.1)

**Table 2:** Central tendency and variability of utility measures

	**% N valid**	**Mean**	**95% CI**	**Median**	**Std. Dev.**	**Minimum**	**Maximum**	**% Floor**	**% Ceiling**
Utilities									
									
Time Trade Off	78.0	72.56	69.43 – 75.70	88.10	31.61	0.00	1.00	2.0	47.3
Standard Gamble	91.1	91.06	89.86 – 92.26	95.00	13.09	0.25	1.00	0.2	47.3

**Table 3:** WTP frequency distribution and descriptive statistics

**WTP**	**(% N)**
0	40.5%
< € 15,000	9.7%
= € 15,000	18.8%
> € 15,000	16.9%
> € 30,000	14.1%

Mean	€ 13,764
Standard deviation	€ 21,719
95% CI	€11,863 – €15,665
Minimum	0
Maximum	€ 100,000
Percentiles	
25	0
50	€ 10,000
75	€ 20,000

**Table 4:** Correlations between utilities and willingness to pay

Utilities	SG	WTP
		
Time Trade Off (TTO)	0.439**	-0.198**
Standard Gamble (SG)		-0.454**

** P < 0.01
